# The prognostic value of POD24 in relapsed/refractory follicular lymphoma—A SCHOLAR‐5 analysis

**DOI:** 10.1002/jha2.1104

**Published:** 2025-02-06

**Authors:** Eve H. Limbrick‐Oldfield, Steve Kanters, Markqayne D. Ray, Timothy Best, Madhu Palivela, Sara Beygi, Anik R. Patel, John G. Gribben, Paola Ghione

**Affiliations:** ^1^ RainCity Analytics Vancouver British Columbia Canada; ^2^ Kite, A Gilead Company Santa Monica California USA; ^3^ Barts Cancer Institute London UK; ^4^ Memorial Sloan Kettering Cancer Center Department of Medicine Lymphoma Service New York New York USA

**Keywords:** follicular lymphoma, non‐Hodgkin lymphoma, POD24, prognostic, real world

## Abstract

**Introduction:**

Follicular lymphoma (FL) has a heterogeneous prognosis. Progression within 24 months of starting front‐line therapy (POD24) is prognostic of overall survival (OS). Despite its prognostic value in early lines, the role of POD24 in relapsed/refractory (R/R) patients initiating later lines of therapy (LoTs) is unknown.

**Methods:**

We analyzed the SCHOLAR‐5 real‐world cohort to investigate whether POD24 is prognostic in patients with R/R FL initiating ≥3rd LoT.

**Results:**

Among the 128 SCHOLAR‐5 patients, 34 patients experienced POD24. POD24 patients received their ≥3 LoT after a shorter time compared with non‐POD24 patients (median 42.0 months [range: 8.8‒17.8] vs. 109.9 months [range: 29.6‒310.2]). Using a time‐dependent multivariate Cox model, POD24 was predictive of shorter OS from initiation of ≥3rd LoT with a hazard ratio (HR) of 2.44 (95% confidence interval [CI]: 1.20‒4.96). For progression‐free survival, using a multivariate repeated‐measures Cox model, the effect was similar but not statistically significant (HR: 1.45; 95% CI: 0.94‒2.11).

**Conclusion:**

This study demonstrates that among patients with R/R FL initiating a ≥3rd LoT, POD24 patients reach these LoTs sooner after diagnosis and POD24 remains prognostic of subsequent OS. This suggests that POD24 status can inform clinical decision making in this population.

## 1 Introduction

Follicular lymphoma (FL) is an indolent form of non‐Hodgkin lymphoma [1, 2]. Despite its indolent nature, FL has a heterogeneous prognosis. Given the varying outcomes in patients with FL, identifying patients that are at higher risk of negative health outcomes using prognostic factors is critical to develop optimal care strategies. One such prognostic factor is progression of disease within 24 months of initiation of front‐line therapy (POD24) [3]. With approximately 20% of patients with FL experiencing POD24 [3], it represents a meaningful portion of patients with FL that may continue to have an unmet medical need [4, 5].

POD24 was first identified as a prognostic variable for FL in a study by Casulo et al. [3]. In this study, patients who underwent POD24 after initiation of front‐line chemoimmunotherapy had shorter subsequent overall survival (OS) following progression, compared with patients who had not progressed by 24 months. Since then, POD24 has been independently validated as a risk factor for shorter OS across several trials and real‐world studies [6, 7, 8] including in a population who received chemotherapy‐free front‐line therapy (e.g., rituximab monotherapy) [9]. Similarly, a more recent validation study by Casulo et al. that included data from 13 clinical trials found that POD24 was prognostic regardless of choice of front‐line therapy, although it had the strongest prognostic impact among patients who received front‐line chemoimmunotherapy [10].

Unlike baseline risk‐factors—such as age, disease stage or the FL international prognostic index (FLIPI) [11]—POD24 cannot be defined until the progression event occurs. As such, it is not available to inform front‐line treatment decisions [12]. Its prognostic value, therefore, lies in treatment selection for subsequent lines in patients with relapsed/refractory (R/R) FL. Given the strong evidence above, POD24 can inform treatment decisions in second‐line settings immediately following initial progression. However, its value at later line of therapy (LoT) is unclear. Despite this, it is often used as a marker for high risk in this population [13, 14]. In the recent pivotal trials of chimeric antigen receptor (CAR) T‐cell therapies and bispecific monoclonal antibodies in patients at ≥3rd LoT, the proportion of patients that previously underwent POD24 has varied from 42% to 63% [15, 16, 17, 18, 19]. This represents a significant proportion of the ≥3rd LoT R/R FL trial population. It is therefore essential to establish if POD24 remains prognostic at ≥3rd LoT.

As FL is an indolent disease, it can take many years to reach ≥3rd LoT. In addition, these patients are a subset of the full FL population, with systematic differences driven by prior disease response and death. A ≥3rd LoT may not be initiated by either the high‐risk patients who die before initiation of this LoT or by the low‐risk patients who respond to earlier LoT and do not relapse. The years between POD24 and initiation of ≥3rd LoT and the change in the characteristics of the patients between these two timepoints may change the prognostic value of POD24. In this study, we sought to investigate POD24 as a prognostic factor in a ≥3rd LoT real‐world cohort, SCHOLAR‐5 [20].

## 2 Methods

### 2.1 Study population

SCHOLAR‐5 is an international real‐world cohort of patients with R/R FL that was initially designed to serve as an external control cohort for the ZUMA‐5 trial [21]. It is comprised of patients recruited in seven medical centers from five countries (United States, UK, France, Spain, and Portugal). Details on the inclusion criteria and characteristics of this cohort have previously been described [20]. In brief, eligible patients were adults (aged ≥ 18 years) with R/R FL grade 1‒3a, with biopsy‐proven absence of transformation, having ≥2 prior LoT and who initiated treatment on or after July 2014. Patients who had prior anti‐CD19 or other CAR T‐cell therapy were ineligible, as were patients who initiated <12 months prior to the study end date (varying by site through 2020).

### 2.2 Definitions

POD24 was defined as progression within 24 months of initiation of front‐line anti‐CD20 containing chemoimmunotherapy. Patients who did not progress by this time, or received other categories of front‐line treatment (e.g., anti‐CD20 monotherapy), were categorized as non‐POD24. Change of therapy within 24 months of initiation without progression was not sufficient to be considered POD24.

Patients could have multiple LoT. All eligible LoT were classed as 3rd, 4th, 5th, or ≥6th in all analyses due to the small number of patients with >6th LoTs. Anti‐CD20 monotherapy was an eligible LoT, but watch and wait, surgery, or radiotherapy alone were not. Refractory disease to prior LoT was defined as disease progression within 6 months of completion of the most recent prior therapy, while relapsed to prior LoT was defined as progression more than 6 months after completion of the most recent prior treatment after complete response, partial response, or stable disease. For OS, the index date was defined as the initiation of the first eligible ≥3rd LoT. Progression‐free survival (PFS) was defined as the time from the index date of each eligible LoT to earliest date of progression or death from any cause. For the description of treatment patterns, all treatments were classified into one of 14 treatment classes (see Supporting Information for more details).

### 2.3 Statistical methods

To assess the association between POD24 and OS among later‐line (i.e., ≥3 LoT) patients with R/R FL, individual patient data from the SCHOLAR‐5 cohort were analyzed using time‐dependent Cox proportional hazards regression [10]. Given the aim of the analysis, we sought to adjust for potential confounders, and as POD24 occurs prior to the initiation of the first eligible ≥3rd LoT, patient characteristics at the start of the first eligible LoT (and therefore after POD24) could not be considered confounders. Instead, we looked to control for patient and disease characteristics at diagnosis, prior to the occurrence (or not) of POD24. The Cox regression was fit with LoT number and stem‐cell therapy (SCT) entered as categorical, time‐varying covariates; and sex and POD24 status were entered as fixed covariates. SCT was included as a binary predictor to indicate which LoT in the data were SCT (including both autologous and allogeneic SCT). As these time‐varying covariates (LoT number and SCT) changed after POD24 status was determined, they were not included as confounders, but rather as predictors to improve modeling performance. Of note, we were unable to include FLIPI and stage at diagnosis due to a high degree of missingness. For OS, the index LoT was the first eligible ≥3rd LoT for each patient. Patients were censored at their last date of follow‐up if they did not have an event. Transformation after initiation of the first eligible LoT was not included as a censoring event as the aim of the analysis was to predict the outcome of eligible patients by POD24 status, and later transformation may be related to this categorization.

In addition to OS, we also explored the prognostic value of POD24 on PFS. These analyses were also conducted using Cox proportional hazards regression; however, changes were made to account for key differences in the outcome definition. Patients can have multiple PFS events—up to one per LoT—while a given patient can only have one OS event. As such, patients were analyzed at each eligible LoT using repeated‐measures mixed‐effects Cox regression. The outcome was time to first progression after initiation of each eligible LoT. The same additional covariates were included as for OS. LoT and SCT were no longer used as time‐varying variables because each LoT was analyzed separately. Ineligible LoTs (such as LoTs following transformation) were excluded.

The proportional hazards assumption was tested for each model by looking for interactions between time residuals for each covariate [22], and the assumption was met in each case. Secondary analyses investigated the relationship between R/R status to the prior LoT to OS and PFS using the same modeling approach for each outcome as described above. These models did not control for POD24 status, due to the collinearity of R/R status and POD24. All statistical analyses used a two‐tailed *α* of 0.05 and were conducted in R (version 4.3.1).

## 3 Results

### 3.1 Patient characteristics

**FIGURE 1:**
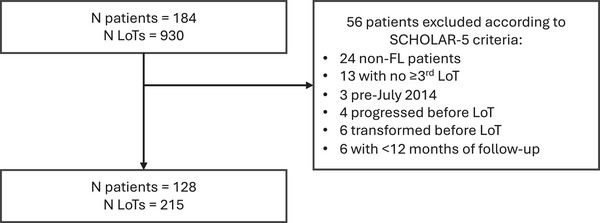
Patient selection. FL, follicular lymphoma; LoT, line of therapy.

**FIGURE 2:**
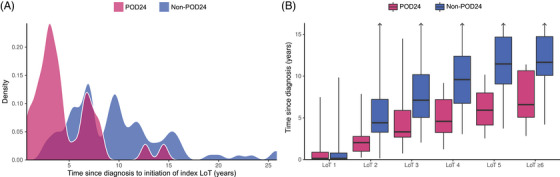
Time course of lines of therapy (LoTs) by progression of disease within 24 months of initiation of front‐line chemoimmunotherapy (POD24). (a) Density plot of time since diagnosis to initiation of first eligible ≥3rd LoT, separated by POD24. (b) Histogram of time since diagnosis to each LoT, separated by POD24. Thick lines represent medians, with the boxes representing the interquartile range (IQR), whiskers representing the range.

Patient selection for the analyses is depicted in Figure 1. Of the 184 recruited patients, 128 met the inclusion criteria for the SCHOLAR‐5 cohort. Within the analysis set, 34 (26.6%) met the criteria for POD24. Table 1 presents and contrasts the baseline characteristics for the eligible patients. Most characteristics at diagnosis were similar between POD24 and non‐POD24 patients. Age, FLIPI, and stage at diagnosis were similar between the two groups. The only statistical difference at diagnosis between the two groups was the year of diagnosis. Non‐POD24 were diagnosed earlier than POD24 patients, with a median year of diagnosis of 2007 and 2013, respectively (Figure S1). This difference may be explained by the shorter survival among POD24 patients that was observed by Casulo et al. Consequently, the median time from diagnosis to initiation of first eligible ≥3rd LoT was significantly longer in the non‐POD24 patients (109.9 months) compared to the POD24 patients (42.0 months, Figure 2), suggesting that POD24 patients have faster progressing disease (i.e., a less indolent form of FL).

**TABLE 1:** Patient characteristics, separated by progression of disease within 24 months of start of front‐line chemoimmunotherapy (POD24) status.

	POD24 (*N* = 34)	Non‐POD24 (*N* = 94)	*p*‐Value^a^
Characteristics at diagnosis			
Median age, years (range)	58 (36, 79)	56 (32, 82)	0.10
FLIPI, *n* (%)			
Low	5 (21.7%)	15 (23.1%)	0.41
Medium	7 (30.4%)	27 (41.5%)	
High	11 (47.8%)	23 (35.4%)	
Missing	11	29	
Stage, *n* (%)			
I	1 (4.0%)	5 (6.7%)	0.81
II	3 (12.0%)	5 (6.7%)	
III	8 (32.0%)	23 (30.7%)	
IV	13 (52.0%)	42 (56.0%)	
Missing	9	19	
Male, *n* (%)	17 (50.0%)	56 (59.6%)	0.44
Characteristics at first eligible ≥3rd LoT			
Median age, years (range)	62 (37, 85)	65.5 (36, 86)	0.48
Median time since diagnosis, months (range)	42.0 (8.8, 173.8)	109.9 (29.6, 310.2)	<0.0001
FL grade, *n* (%)			
Grade 1	13 (39.4%)	46 (54.8%)	0.04
Grade 2	13 (39.4%)	33 (39.3%)	
Grade 3a	7 (21.2%)	5 (6.0%)	
Missing	1	9	
ECOG, *n* (%)			
≤1	27 (96.4%)	67 (91.2%)	0.27
>1	1 (3.6%)	6 (8.2%)	
Missing	6	21 (91.8%)	
Number of prior LoT, median (range)	2 (2‒4)	2 (2‒9)	<0.01
Response to previous LoT			
Relapsed	16 (47.1%)	63 (68.5%)	0.05
Refractory	18 (52.9%)	29 (31.5%)	
Missing	0	2	
Double refractory, *n* (%)	9 (26.5%)	3 (3.2%)	<0.0001
Size of largest nodal mass ≥7 cm, *n* (%)	7 (31.8%)	15 (25.0%)	0.74
Missing	12	34	

Abbreviations: ECOG, Eastern Cooperative Oncology Group performance score; FL, follicular lymphoma; FLIPI, follicular lymphoma international prognostic index; LoT, line of therapy.

^a^ Characteristics were compared between groups using chi‐square test (for categorical outcomes) or an independent *t*‐test (for continuous outcomes). Time since diagnosis and number of prior LoT did not meet the assumption of normality, and so groups were compared using a Mann‒Whitney *U*‐test.

**FIGURE 3:**
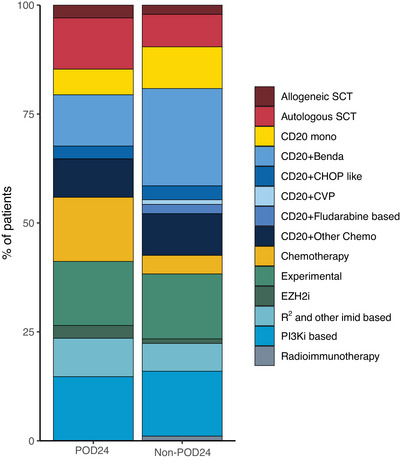
Treatment patterns at first eligible line of therapy (LoT). Benda, bendamustine; CD20; anti‐CD20 monoclonal antibodies; Chemo, chemotherapy; CHOP, cyclophosphamide, doxorubicin, vincristine, and prednisone; CVP, cyclophosphamide, vincristine, prednisolone; EZH2i, enhancer of zeste homolog 2‐specific inhibitors, IMiD, immunomodulatory drugs; R^2^, rituximab and lenalidomide; SCT, stem cell transplant; PI3Ki, phosphoinositide 3‐kinase inhibitor; POD24, progression of disease within 24 months of front‐line chemoimmunotherapy.

At this first eligible ≥3rd LoT, POD24 patients were also significantly more likely to be double refractory (defined as refractory to the two LoTs prior to the index LoT), have higher grade FL, and shorter median time since diagnosis, as presented in Table 1. Although the median number of prior lines was two for both groups, the range of prior LoTs was much wider in the non‐POD24 group, with a maximum of nine prior LoTs, compared to a maximum of four in the POD24 group. Treatment patterns at first eligible ≥3rd LoT were similarly heterogenous in POD24 and non‐POD24 patients (Figure 3 and Table S1), as were treatment patterns across all LoT (Figure S2). We did observe, however, that fewer POD24 patients received anti‐CD20 monotherapy at second LoT, compared to non‐POD24 patients.

### 3.2 Effectiveness outcomes

**FIGURE 4:**
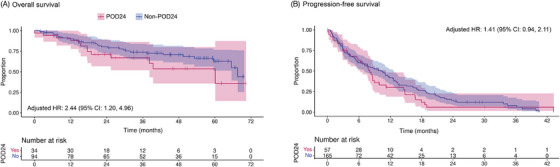
Unadjusted Kaplan‒Meier curves by progression of disease within 24 months of start of front‐line chemoimmunotherapy (POD24) status. CI, confidence interval; HR, hazard ratio; LoT, line of therapy.

Results of the Cox proportional hazards regression models are presented in Table 2 (full details in Table S2). POD24 was a statistically significant predictor of OS (hazard ratio [HR]: 2.44; 95% confidence interval [CI]: 1.20‒4.96). POD24 patients initiating at ≥3rd LoT were at 2.44 times higher risk of death than their non‐POD24 counterparts. The unadjusted Kaplan‒Meier curve (Figure 4a) also shows this pattern of results, despite this plot not controlling for the effect of the confounders included in the model. As previously mentioned, patients were not censored at transformation of disease. A review of the data revealed that only a small proportion of patients showed evidence of transformation before the end of follow‐up (Figure S3). Patients that showed evidence of transformation prior to their first eligible LoT were not included in the SCHOLAR‐5 cohort.

**TABLE 2:** Survival estimates by progression of disease within 24 months of front‐line chemoimmunotherapy (POD24).

	POD24	Non‐POD24
Overall survival		
Adjusted HR (95% CI)	2.44 (1.20, 4.96)	
Median months (95% CI)	60.1 (38.2, NE)	67.6 (65.5, NE)
24 months, % (95% CI)	71.1 (56.8, 89.1)	80.3 (72.3, 89.2)
Progression‐free survival		
Adjusted HR (95% CI)	1.41 (0.94, 2.11)	
Median months (95% CI)	8.1 (6.2, 11.2)	10.3 (7.1, 12.6)
24 months, % (95% CI)	6.0 (1.6, 22.7)	14.4 (8.9, 23.4)

Abbreviations: CI, confidence interval; HR, hazard ratio; NE, not evaluable.

In the PFS model that included all eligible LoTs (also presented in Table 2), the effect was in the same direction as OS but not statistically significant. The lower bound of the estimated treatment effect was close to 1, which suggests marginal support for the value of POD24 as a prognostic factor for PFS. Nonetheless, the estimated effect was also considerably smaller than that observed in OS (HR: 1.41; 95% CI: 0.94‒2.11, Figure 3c).

Additional analyses provided further insights into the data. R/R status to prior LoT (Figure S4 and Table S3) was a not a significant predictor of OS (HR: 1.39; 95% CI: 0.71‒2.72) but was a predictor of PFS (HR: 1.50; 95% CI: 1.05‒2.14). Finally, the POD24 group did not contain any patients initiating ≥5th LoT, whereas there were seven such patients in the non‐POD24 group. The results remained unchanged when the analysis was conducted without these seven patients.

## 4 Discussion

Our research expands upon the pivotal work by Casulo et al. [3, 10] by exploring whether POD24 remains prognostic of OS in patients initiating later LoTs in R/R FL. This study demonstrates that POD24 retains its prognostic value for OS among later‐line patients with R/R FL, with POD24 patients experiencing poorer OS after initiation of ≥3rd LoT. In addition, the time to reach these later lines was much shorter for patients with POD24, which suggests that these patients progress through subsequent LoTs faster than those who do not undergo POD24. This progression early in the disease course has an effect on the treatment pathway that remains significant at later LoT.

OS has been the outcome of interest in prior investigations of POD24 [3, 10, 23] likely due to its objectivity and clinical meaningfulness [24], as well as its availability across multiple trials and real‐word sources. However, PFS is also an important outcome in FL studies, as it requires considerably less follow‐up time to reach maturity. Here, we did observe an effect in the expected direction for PFS, with POD24 being associated with shorter PFS among later‐line patients with R/R FL. However, as the association to PFS was not statistically significant, this relationship is worth subsequent investigations in other, potentially larger, cohorts. Further work is needed to establish if this lack of significance reflects the true absence of an effect of POD24 on PFS, or if we were lacking the statistical power to observe the effect in our sample.

The definitions used for POD24 in this later‐line population are heterogeneous, particularly with regards to the front‐line therapy patients received to be considered POD24 after a progression event. In our study, we used the ZUMA‐5 definition: progressing within 24 months of the start of front‐line chemoimmunotherapy with patients who received front‐line monotherapies being classified as non‐POD24 [17]. This definition was also used by the TRANSCEND‐FL trial for lisocabtagene maraleucel, but differs from the recent analysis of Casulo et al., where patients who progressed following frontline monotherapy were also considered POD24 [16]. The definition used in the ELARA differed again [18], with patients considered POD24 as long as front‐line therapy included an anti‐CD20 monoclonal antibody (i.e., rituximab monotherapy was eligible). In addition, changing to second‐line therapy, regardless of disease progression, was an eligible event. In the GO29871 trial for mosunetuzumab, patients were considered POD24 regardless of the type of systemic front‐line therapy administered [15]. The definition used in our analysis was motivated by earlier work in POD24 that suggested a strong effect of POD24 among patients initiating front‐line chemoimmunotherapy [3]. Since this time, additional investigations have shown that early progression after front‐line treatment with less intense regimens (e.g., rituximab monotherapy) may also be prognostic [9, 10]. However, whichever definition is used, patients who receive more aggressive front‐line therapy and still progress early meet the criteria for POD24, and so all these definitions capture the highest risk population.

The SCHOLAR‐5 cohort was designed to be representative of relatively recent treatment regimens. To this end, all eligible LoTs in this cohort were initiated after July 2014. The treatment pattern looked similar in POD24 patients, except for first‐line treatment as this was used in the POD24 definition. There is no evidence that there was a systematic difference in the type of therapy POD24 patients received at later LoTs (e.g., both groups received similar rates of experimental treatment), and no evidence that treatment differences at index LoT could explain the difference in OS. As such, controlling for SCT, as we did in the analysis, was likely sufficient to adjust for potential bias that may result from differences in treatment patterns [25]. As SCHOLAR‐5 was designed as an external control arm for ZUMA‐5, no SCHOLAR‐5 patients received CAR T‐cell therapy, so no further analytical adjustments were required. It is noteworthy, however, that we have previously shown that the clinical benefit of axicabtagene ciloleucel, when comparing ZUMA‐5 to SCHOLAR‐5, was maintained in the POD24 subgroup analysis [21].

Our study has some limitations. First, due to the indolent nature of R/R FL, the low proportion of patients with FL that initiate a ≥3rd LoT, and the requirement that patients must have initiated an eligible LoT after July 2014, the sample size of this cohort can be qualified as modest. Similar sampling challenges were observed in other similar external control cohort studies [26, 27, 28]. Although our study may have been underpowered for detecting a relationship to PFS, we did observe a statistically significant prognostic relationship of POD24 for OS. In addition to the small sample size, the patients included in the study were heterogenous in terms of prior treatment and the number of prior LoT. SCHOLAR‐5 does not include recently approved treatments, such as CAR T‐cell therapies and bispecific monoclonal antibodies, and the prognostic value of POD24 in these interventions is not known. Whilst this cohort does include patients from both the United States and Europe, further work would be required to investigate if the prognostic value of POD24 is maintained outside of these geographies. Finally, some baseline characteristics were adjusted for in previous POD24 research that we were not able to include due to missingness. Given that this research was focused on later LoTs, it is not clear how impactful patient characteristics at diagnosis would impact these analyses. The impact of patient characteristics at diagnosis would likely be less impactful over time.

In this cohort, the number of patients with evidence of transformation after initiation of the first eligible LoT was minimal, although there were more patients in the POD24 subgroup who transformed during follow‐up. This may suggest that POD24 is a risk factor for later transformation, although more data would be required to investigate this. As patients who transformed prior to their first eligible LoT were excluded from SCHOLAR‐5 this reduced the size of the POD24 population and removed those patients with the most aggressive disease [29]. This may have given a more accurate estimate of the effect on OS, compared to other studies where transformation was not accounted for.

Prognostic factors with strong effects are particularly important to identify in FL given that identifying patients with less indolent disease may help healthcare providers optimize patient care. Our study has shown that POD24 continues to hold strong prognostic value in later‐line patients with R/R FL and is likely to have prognostic value for other outcomes, including PFS. More research is needed to better understand the prognostic value of POD24 on other outcomes, the impact of the heterogeneous definitions found across this therapeutic area, and in understanding whether its prognostic value holds for patients using more modern treatments such as CAR T‐cell therapies.

## Conflicts of Interest

Eve H Limbrick‐Oldfield and Steve Kanters, current employment at RainCity Analytics; Markqayne D Ray, Timothy Best, Madhu Palivela, Sara Beygi, Anik R Patel, current employment at and holding stock and stock options from Kite, a Gilead company; John Gribben, reports receiving consulting fees from AbbVie, Acerta Group Limited/AstraZeneca, Bristol Myers Squibb/Celgene Corporation, Janssen, Kite/Gilead, research funding from Acerta Group Limited/AstraZeneca, Bristol Myers Squibb/Celgene Corporation, and Janssen; Paola Ghione reports no conflict of interest.

## Clinical Trial Registration

The authors have confirmed clinical trial registration is not needed for this submission.

## Supporting information



Supporting information

## Data Availability

All data are confidential. They can be made available upon approval of a research proposal and signed data access agreement.
