# Supplementary material for: The prognostic value of POD24 in relapsed/refractory follicular lymphoma—A SCHOLAR‐5 analysis
Source: EJHaem. 2025 Feb 6;6(1):e1104. doi: 10.1002/jha2.1104 (PMC11800366; doi:10.1002/jha2.1104)
Supplement: Supplementary file 1 — Supporting information [file JHA2-6-e1104-s001.docx]

| **The prognostic value of POD24 in relapsed/refractory follicular lymphoma – A SCHOLAR-5 analysis.** |
| --- |
| *Supplementary materials* |

**Contents**

[Supplemental methods 2](#_Toc176425593)

[Figure S1: Date of diagnosis by POD24: a) histogram and b) density plot 3](#_Toc176425594)

[Table S1: Treatment patterns for index LoT 4](#_Toc176425595)

[Figure S2: Treatment patterns alluvial plots comparing POD24 and non-POD24 patients 5](#_Toc176425596)

[Figure S3: Number of patients who showed transformation of disease prior to each LoT 6](#_Toc176425597)

[Figure S4: Unadjusted Kaplan-Meier curves by relapsed / refractory to prior LoT 7](#_Toc176425598)

Supplemental methods

*Treatment categories*

For analytic purposes, treatment regimens were grouped into the following categories to ease interpretation of results: allogeneic SCT, autologous SCT, anti-CD20 monoclonal antibody monotherapy, anti-CD20 monoclonal antibodies plus bendamustine (CD20+Benda), CD20+CHOP (rituximab, cyclophosphamide, doxorubicin hydrochloride, vincristine and prednisone) like, CD20+CVP (cyclophosphamide, vincristine and prednisone), CD20+fludarabine based, CD20+other chemo, chemotherapy (other), experimental, EZH2i (enhancer of zeste homolog 2 specific inhibitors), R2 (rituximab and lenalidomide) and other imid-based, PI3K inhibitor based and radioimmunotherapy. The CHOP-like category included primarily CHOP, but also CHEP (etoposide instead of vincristine) and EPOCH (CHOP + etoposide). Other chemotherapy primarily included platinum-based chemotherapies and chlorambucil, but also included a variety of others. Experimental treatments included treatments described as experimental treatments or considered off-label. They included SYK-inhibitors, PD1-inhibitors, and BCL2-inhibitors, among others.

Figure S1: Date of diagnosis by POD24: a) histogram and b) density plot

a) b)

**
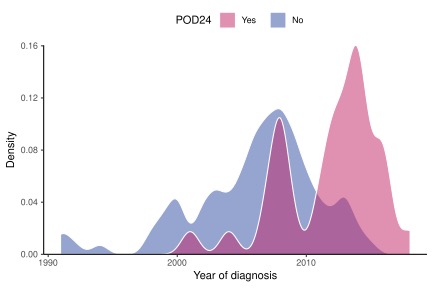
**

*POD24: progression of disease within 24 months of front-line chemo-immunotherapy.*

Table S1: Treatment patterns for index LoT

|  | **POD24  (N = 34)** | **Non POD24  (N = 94)** |
| --- | --- | --- |
| Allogeneic SCT | 1 (2.9%) | 2 (2.1%) |
| Autologous SCT | 4 (11.8%) | 7 (7.4%) |
| CD20 mono | 2 (5.9%) | 9 (9.6%) |
| CD20 + Benda | 4 (11.8%) | 21 (22.3%) |
| CD20 + CHOP like | 1 (2.9%) | 3 (3.2%) |
| CD20 + CVP |  | 1(1.1%) |
| CD20 + Fludarabine based | 0 | 2 (2.1%) |
| CD20 + Other Chemo | 3 (8.8%) | 9 (9.6%) |
| Chemotherapy | 5 (14.7%) | 4 (4.3%) |
| Experimental | 5 (14.7%) | 14 (14.9%) |
| EZH2i | 1 (2.9%) | 1 (1.1%) |
| R^2^ and other imid based | 3 (8.8%) | 6 (6.4%) |
| PI3Ki based | 5 (14.7%) | 14 (14.9%) |
| Radioimmunotherapy | 0 | 1 (1.1%) |

*Number (and percent) of patients that received each treatment category at the index LoT for the analysis of overall survival. Benda; bendamustine; CD20; anti CD20 monoclonal antibodies; Chemo: chemotherapy; CHOP: cyclophosphamide, doxorubicin, vincristine, and prednisone; CVP: cyclophosphamide, vincristine, prednisolone; EZH2i: Enhancer of zeste homolog 2 specific inhibitors, IMiD: immunomodulatory drugs; LoT: line of therapy; R^2^: rituximab and lenalidomide; SCT: stem cell transplant; PI3Ki: phosphoinositide 3-kinase inhibitor; POD24: progression of disease within 24 months of front-line chemo-immunotherapy.*

Table S2: Results of adjusted analyses (POD24)

| Predictor | HR | 95% CI |
| --- | --- | --- |
| OS (POD24 model adjusted for LoT, Sex and SCT) | | |
| POD24 (Yes) | 2.44 | 1.20, 4.96 |
| LoT 4 | 0.90 | 0.30, 2.66 |
| LoT 5 | 2.84 | 1.00, 8.09 |
| LoT ≥6 | 10.54 | 4.47, 24.88 |
| Sex (Female) | 0.93 | 0.50, 1.72 |
| Current LoT SCT (Yes) | 0.81 | 0.28, 2.38 |
| PFS (POD24 model adjusted for LoT, Sex and SCT) | | |
| POD24 (Yes) | 1.41 | 0.94, 2.11 |
| LoT 4 | 1.27 | 0.85, 1.90 |
| LoT 5 | 1.88 | 1.10, 3.21 |
| LoT ≥6 | 2.74 | 1.58, 4.76 |
| Sex (Female) | 1.09 | 0.76, 1.57 |
| Current LoT SCT (Yes) | 0.69 | 0.39, 1.22 |

LoT was entered as a categorical predictor, all levels compared to LoT 3.

Table S3: Results of adjusted analyses (Relapsed / Refractory status)

| Predictor | HR | 95% CI |
| --- | --- | --- |
| OS | | |
| Refractory | 1.39 | 0.71, 2.72 |
| LoT 4 | 0.81 | 0.27, 2.39 |
| LoT 5 | 2.11 | 0.76, 5.85 |
| LoT ≥6 | 8.08 | 3.51, 18.60 |
| Sex (Female) | 1.05 | 0.56, 1.99 |
| Current LoT SCT (Yes) | 0.74 | 0.25, 2.18 |
| PFS | | |
| Refractory | 1.50 | 1.05, 2.14 |
| LoT 4 | 1.18 | 0.78, 1.79 |
| LoT 5 | 1.67 | 1.00, 2.80 |
| LoT ≥6 | 2.13 | 1.18, 3.82 |
| Sex (Female) | 1.08 | 0.75, 1.54 |
| Current LoT SCT (Yes) | 0.59 | 0.31, 1.09 |

LoT was entered as a categorical predictor, all levels compared to LoT 3.

Figure S2: Treatment patterns alluvial plots comparing POD24 and non-POD24 patients

a) POD24 patients

b) Non-POD24 patients

*Treatment patterns in all LoTs from eligible patients, separated by POD24 status. Shaded areas indicate transition between treatment categories between LoTs. Second line therapy was autologous SCT for 7/34(20.6%) of POD24 patients and 14/94 (14.9%) of non POD24 patients. No patients received allogeneic SCT at second line.*  *Benda; bendamustine; CD20; anti CD20 monoclonal antibodies; Chemo: chemotherapy; CHOP: cyclophosphamide, doxorubicin, vincristine, and prednisone; CVP: cyclophosphamide, vincristine, prednisolone; EZH2i: Enhancer of zeste homolog 2 specific inhibitors, IMiD: immunomodulatory drugs; LoT: line of therapy; R^2^: rituximab and lenalidomide; SCT: stem cell transplant; PI3Ki: phosphoinositide 3-kinase inhibitor; POD24: progression of disease within 24 months of front-line chemo-immunotherapy*

Figure S3: Number of patients who showed transformation of disease prior to each LoT

a) POD24 patients

b) Non-POD24 patients


*Patients were included in the overall survival analysis if there was no evidence of transformation prior to their index LoT, but were not censored at transformation. For the analysis of progression-free survival, only LoTs without evidence of transformation were included in the model. LoT: line of treatment; POD24: progression of disease within 24 months of front-line chemo-immunotherapy.*

Figure S4: Unadjusted Kaplan-Meier curves by relapsed / refractory to prior LoT

a) Overall survival b) Progression-free survival


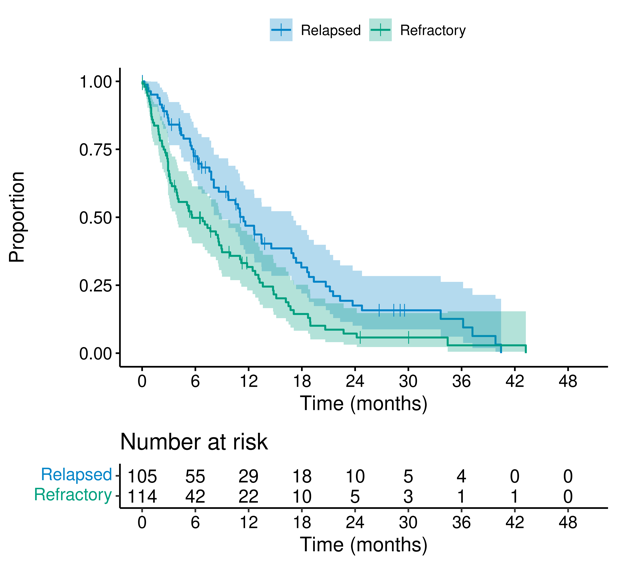


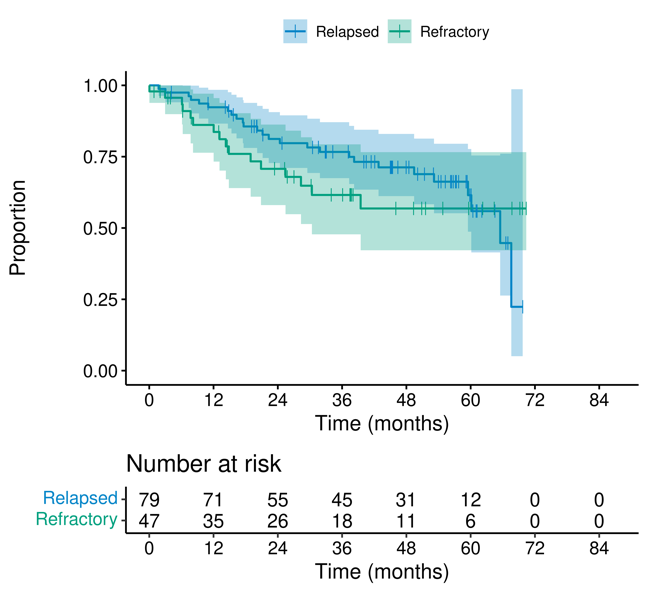


*a) Adjusted hazard ratio for overall survival: 1.30 (95% confidence interval: 0.67, 2.54).
b) Adjusted hazard ratio for progression-free survival: 1.50 (95% confidence interval: 1.05, 2.14).*
